# Non-professional marathon running: RAGE axis and ST2 family changes in relation to open-window effect, inflammation and renal function

**DOI:** 10.1038/srep32315

**Published:** 2016-09-22

**Authors:** Christine Bekos, Matthias Zimmermann, Lukas Unger, Stefan Janik, Philipp Hacker, Andreas Mitterbauer, Michael Koller, Robert Fritz, Christian Gäbler, Mario Kessler, Stefanie Nickl, Jessica Didcock, Patrick Altmann, Thomas Haider, Georg Roth, Walter Klepetko, Hendrik Jan Ankersmit, Bernhard Moser

**Affiliations:** 1 Christian Doppler Laboratory for Cardiac and Thoracic Diagnosis and Regeneration, Medical University Vienna, Austria; 2 Department of Obstetrics and Gynaecology, Division of Gynaecology and Gynecological Oncology, Medical University Vienna, Austria; 3 Sportordination, Alserstraße 27/1/6, Vienna, Austria; 4 Austrian Red Cross, Nottendorfer Gasse 21, 1030 Wien, Austria; 5 Department of Surgery, Division of Plastic and Reconstructive Surgery, Medical University Vienna, Austria; 6 Department of Trauma Surgery, Medical University Vienna, Austria; 7 Department of Anesthesia, Critical Care and Pain Medicine, Medical University Vienna, Austria; 8 Department of Surgery, Division of Thoracic Surgery, Medical University Vienna, Austria

## Abstract

Conflicting data exist on the relevance of marathon (M) and half marathon (HM) running for health. The number of non-professional athletes finishing M and HM events is steadily growing. In order to investigate molecular changes occurring in amateur athletes, we enrolled 70 non-professional runners finishing a single M (34) or HM (36) event at baseline, the finish line and during recovery, and 30 controls. The measurement of the Receptor for Advanced Glycation Endproducts, Interleukin 1 receptor antagonist, ST2 and cytokeratin 18 was combined with molecules measured during clinical routine. Results were analyzed in the light of blood cell analysis, lactate measurements, correction for changes in plasma volume and body composition assessments. There were intrinsic differences in body mass index, abdominal body fat percentage and training time between M and HM runners. C-reactive protein changes in M and HM runners. While soluble RAGE, AGEs and ST2 increased immediately after the race in HM runners, HMGB1 increased in HM and M after the race and declined to baseline after a recovery period. We give insights into the regulation of various molecules involved in physical stress reactions and their possible implications for the cardiovascular system or renal function.

In the United States a 32% increase in marathon (M) finishers and a 307% increase in half marathon (HM) finishers was observed over the last decade^1^,^2^ and therefore resembles increasing popularity.

Regular moderate endurance training has been shown to be effective in prevention of obesity and hypertension^3^, diabetes^4^, osteoporosis^5^, cancer^6^ and aging^7^. Running was reported to reduce all-cause and cardiovascular mortality by 30–45% with an increase in life expectancy by three years in a study conducted with 55000 adults that were followed for 15 years^8^. However, the benefits of running were less pronounced in subjects running more than twelve miles and more than three times per week, with an exercise duration longer than 120 minutes per week as well as with a pace faster than seven miles per hour^8^. These results emphasized the finding that excessive training might impair health rather than promoting it.

Although the positive effects of endurance training itself are well documented, data is scarce regarding eventual health benefits to the participation of single M or HM events. Observations of sub-clinical coronary artery disease and subclinical myocardial fibrosis that increased by the number of completed M are conflicting the sole beneficial effects^9^. Performing coronary angiography in 50 males participating in at least one M yearly for 25 consecutive years, a significantly higher accumulation of hard and soft coronary plaques was observed when compared to sedentary controls^10^.

In summary, although chronic endurance training seems to have protective effects on the telomere length and therefore might decelerate the biological aging process, participation in M races may lead to telomere shortening caused by oxidative DNA damage^7^ and benefits of running are hampered by “overdosing” of exercise. These conflicting results raise the question whether a single stressfull event such as running a long-distance run can lead to activation of cell-stress specific molecular programs that are also activated during illness and disease.

Marathon running can result in damage to muscle cells which can lead to a transient rise in serum creatinine that meets criteria of AKI^11^. These elevations resolve within 24 h without obvious consequences. The impact of repetitive episodes of AKI associated with long-distance running on long-term kidney function remains unknown.

A vast literature exists on the physiological and pathophysiological role of the Receptor of Advanced Glycation Endproducts (RAGE) axis, interleukin (IL)-1 receptor family receptor suppression of tumorigenicity 2 (ST2) and its ligand IL33 in cardiovascular disease^12^, cancer^13^ and autoimmune diseases^14^. However, data is insufficient when it comes to healthy subjects in extraordinary situations such as extensive physical activity.

RAGE is a member of the immunoglobulin superfamily. Apart from advanced glycation endproducts (AGEs), various ligands such as high mobility group box 1 (HMGB1) were observed to interact with this cell surface receptor. Soluble RAGE (sRAGE) can either be a splice variant of RAGE which is actively secreted, termed endogenous secretory RAGE (esRAGE) or can be proteolytically cleaved from the cell surface. SRAGE acts as a decoy receptor binding circulating ligands and therefore prevents membrane-bound RAGE from activation. A dual function of the RAGE family has not only been described in chronic diseases such as atherosclerosis^15^, asthma^16^, sepsis^17^, diabetes^18^ and cancer^13^,^19^ but also in physiology^20^. RAGE is known to play a role in the development and course of kidney diseases regardless of the etiology^21^. Further, increased soluble RAGE concentrations have been observed in the progressive impairment of renal function^22^ and is suggested as a potential biomarker for renal insufficiency^23^. To date, there is no published literature focusing on the effects of a single endurance event on serum concentrations of sRAGE, esRAGE and RAGE ligands in amateur runners.

IL33 is a member of the IL-1 cytokine family, acts in an anti-inflammatory and anti-angiogenetic manner and is the ligand of ST2. Soluble ST2 (sST2) acts as a decoy receptor. IL33 and ST2 were reported to be regulated in response to myocardial stress, in asthma, rheumatoid arthritis, collagen vascular diseases, sepsis, trauma, malignancy and ulcerative colitis^24^. Next to troponin, ST2 is already used in clinical routine to predict mortality in patients with heart failure and for monitoring the medical therapy^25^.

Interleukin 1 receptor antagonist (IL1-RA) deficiency has been shown to increase susceptibility for and microbial colonisation in patients with cystic fibrosis. This effect can be antagonised by administration of the recombinant IL-1Ra, anakinra^26^.

During apoptosis cytokeratin (CK)18 is cleaved into proteolytic fragments by caspase 3, 6 and 7 and by then is called caspase-cleaved CK18 (ccCK18)^27^. CK18 is known to function as an intracellular scaffold protein, to resist cell stresses and to maintain mitochondrial structures and cellular processes such as apoptosis, mitosis, cell cycle progression, and cell signalling^28^. Therefore, in apoptosis ccCK18 is increased while the level of necrosis can be assessed by calculating the margin between ccCK18 and total CK18. CK18 has been implicated in chronic kidney disease^29^ and in nonalcoholic fatty liver disease for distinguishing nonalcoholic steatohepatitis from simple steatosis^30^. In the present study we tried to gain a better understanding of pro- and anti-inflammatory processes happening during extraordinary physical activity. The findings were analysed in the light of using well-established surrogate markers that are routinely available.

## Results

### Demographic data

Thirty-four marathoners, 36 half-marathoners and 30 sedentary volunteers were included in this study. Basic demographic data are listed in Table 1. We found statistically significant differences in BMI, abdominal girth and body fat percentage, when marathoners, half-marathoners and sedentary controls were compared. Post hoc analysis revealed lower BMI, abdominal girth and body fat in marathoners. There were no significant differences in age, female:male ratio, weight, hip size and smoking habits between the three groups. For description of smoking habits the number of Smoker:Nonsmoker:Neversmoker was 01:08:25 for the M runners, 02:06:28 for HM runners and 03:04:23 for the controls. The number of pack years (PY) was 1.70 ± 0.85 PY in marathoners, 3.23 ± 1.28 half-marathoners and 3.32 ± 1.86 in the control group (Table 1).

### Running time and training distance

The M time of study cohort was 226.8 ± 5.3 minutes (min) and the HM time was 117.0 ± 2.8 min, p < 0.001. Marathoners trained for 4.5 ± 0.2 hours per week, half-marathoners for 3.5 ± 0.2 hours per week, p = 0.001. The weekly running distance in marathoners was 61.85 ± 3.96 km compared to 35.10 ± 2.64 km in half-marathoners, p < 0.001 (Table 2).

### Changes in plasma volume

The mean change in plasma volume (%) that occurred in M and HM runners was 1.89 ± 1.88 and −5.38 ± 4.63. Therefore all of the results presented below are corrected for changes in plasma volume.

### White and red blood cell analysis

Results of white and red blood cell analysis are detailed in supplemental material.

Red blood cell counts (RBC) were significantly increased after the race, both in M (M_baseline vs. M_peak, p = 0.008) and HM runners (HM_baseline vs. HM_peak, p = 0.027) and returned to baseline after 2 to 7 days of recovery.

Hemoglobin (HGB) rose significantly after running a HM (HM_baseline vs. HM_peak, p = 0.007) and returned to baseline levels after one week of recovery. We could not detect any significant changes in M runners.

There were no differences in hematocrit (HCT) concentrations detectable.

The mean corpuscular volume (MCV) increased significantly after running a M or HM (M_baseline vs. M_peak, p < 0.001; HM_baseline vs. HM_peak, p < 0.001) and both returned to baseline levels again after 2 to 7 days of recovery.

The mean corpuscular hemoglobin (MCH) increased significantly after running a M or HM (M_baseline vs. M_peak, p < 0.001; HM_baseline vs. HM_peak, p < 0.001) and both returned to baseline levels again after 2 to 7 days of recovery.

The mean corpuscular hemoglobin concentration (MCHC) increased significantly after running a M or HM (M_baseline vs. M_peak, p < 0.001; HM_baseline vs. HM_peak, p < 0.001) and both returned to baseline levels again after 2 to 7 days of recovery.

**Figure 1:**
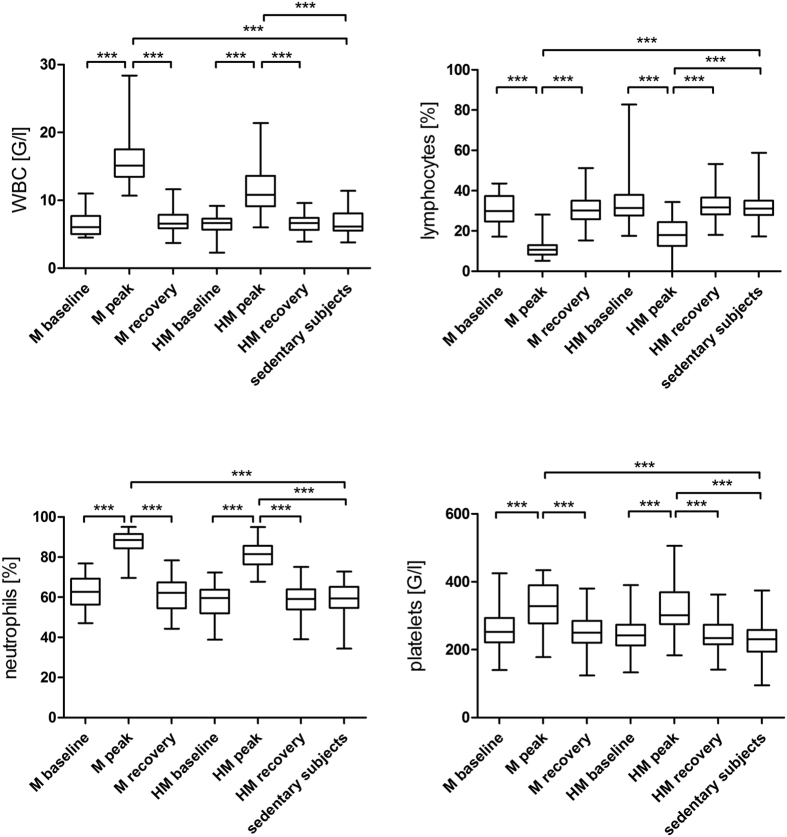
(**a**) White blood cell count in marathoners and half-marathoners. White blood cell counts significantly increased after the race, both in M and HM and returned to baseline after 2 to 7 days of recovery. M, marathon; HM, half-marathon; baseline, 1–2 days before the run; peak, immediately after the run in the finishing area; recovery, after 2–7 days of recovery; WBC, white blood cell counts. ***p < 0.001. (**b**) Lymphocytes in percent of leucocytes in marathoners and half-marathoners. Lymphocytes significantly decreased after the race, both in M and HM runners. M, marathon; HM, half-marathon; baseline, 1–2 days before the run; peak, immediately after the run in the finishing area; recovery, after 2–7 days of recovery; ***p < 0.001. (**c**) Neutrophils in percent of leucocytes in marathoners and half-marathoners. Neutrophils significantly increased after the race, both in M and HM runners. M, marathon; HM, half-marathon; baseline, 1–2 days before the run; peak, immediately after the run in the finishing area; recovery, after 2–7 days of recovery; ***p < 0.001. (**d**) Platelets in marathoners and half-marathoners. Platelets significantly increased after the race, both in M and HM runners. M, marathon; HM, half-marathon; baseline, 1–2 days before the run; peak, immediately after the run in the finishing area; recovery, after 2–7 days of recovery; ***p < 0.001.

White blood cell (WBC) counts were significantly increased after the race, both in M (M_baseline vs. M_peak, p < 0.001) and HM runners (HM_baseline vs. HM_peak, p < 0.001) and returned to baseline after 2 to 7 days of recovery (Fig. 1a).

Immediately after participating in M or HM lymphocytes decreased significantly (M_baseline vs. M_peak, p < 0.001, HM_baseline vs. HM_peak, p < 0.001), but climbed up to baseline levels again on after 2 to 7 days of recovery (Fig. 1b).

Monocytes decreased significantly in HM runners immediately after the race (HM_baseline vs. HM_peak, p = 0.001) and returned to baseline levels after 2 to 7 days of recovery. No changes were observed in M runners.

Neutrophils increased immediately after running a M (M_baseline vs. M_peak, p < 0.001) or a HM (HM_baseline vs. HM_peak, p < 0.001) but returned to baseline levels again after one week of recovery (Fig. 1c).

### Platelet analysis

Platelet counts (PLT) rose significantly after running a M or HM (M_baseline vs. M_peak, p < 0.001, HM_baseline vs. HM_peak, p < 0.001) and returned to baseline levels after one week (Fig. 1d).

The mean platelet volume (MPV) increased significantly after running a M or HM (M_baseline vs. M_peak, p = 0.001; HM_baseline vs. HM_peak, p = 0.005) and both returned to baseline levels again after 2 to 7 days of recovery.

### Anaerobic metabolism

**Figure 2:**
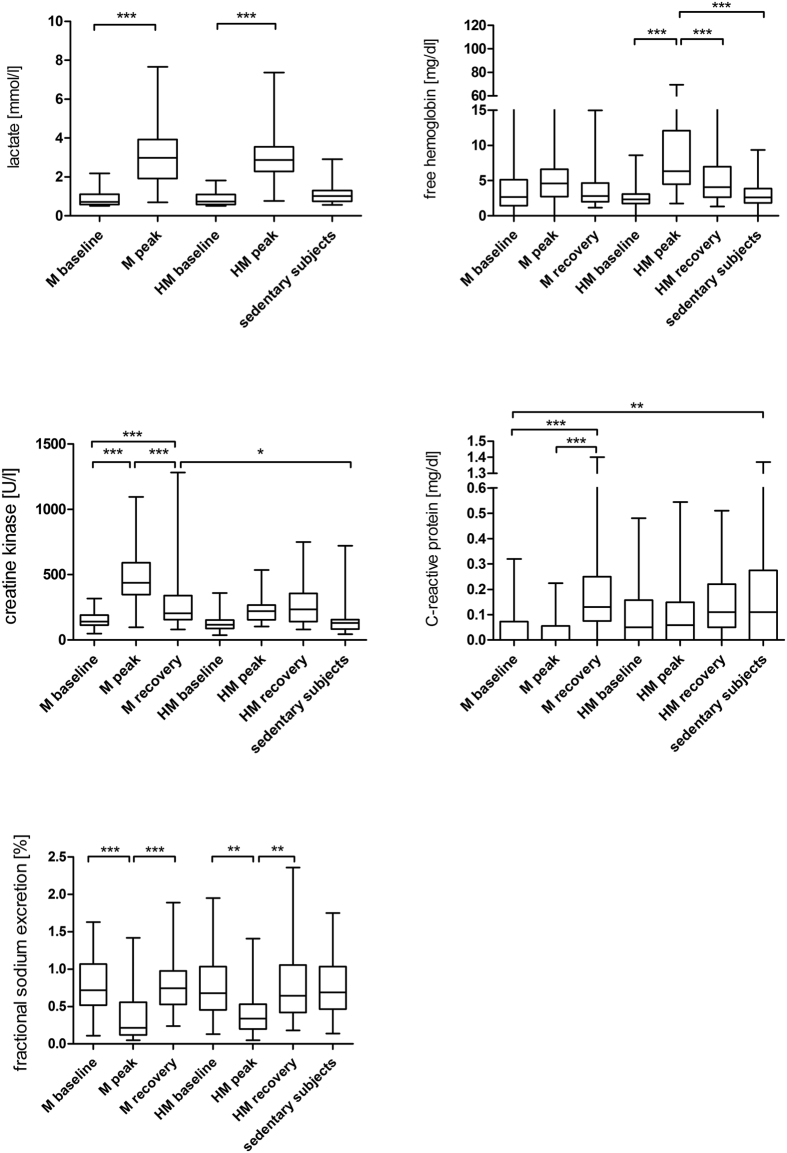
(**a**) Lactate in marathoners and half-marathoners. Lactate significantly increased after the race, both in M and HM runners. M, marathon; HM, half-marathon; baseline, 1–2 days before the run; peak, immediately after the run in the finishing area; ***p < 0.001. (**b**) Free hemoglobin in marathoners and half-marathoners. Free hemoglobin significantly increased after the race only in half-marathoners. M, marathon; HM, half-marathon; baseline, 1–2 days before the run; peak, immediately after the run in the finishing area; recovery, after 2–7 days of recovery; ***p < 0.001. (**c**) Creatine kinase in marathoners and half-marathoners. Creatine kinase increased significantly after the race in M runners only and remained significantly above baseline concentrations at 2 to 7 days of recovery. M, marathon; HM, half-marathon; baseline, 1–2 days before the run; peak, immediately after the run in the finishing area; recovery, after 2–7 days of recovery; *p < 0.05, ***p < 0.001. (**d**) C-reactive protein (CRP) in marathoners and half-marathoners. CRP was significantly lower in marathoners at rest than in sedentary subjects and increased after 2 to 7 days of recovery. M, marathon; HM, half-marathon; baseline, 1–2 days before the run; peak, immediately after the run in the finishing area; recovery, after 2–7 days of recovery; **p < 0.01, ***p < 0.001. (**e**) Fractional sodium excretion in marathoners and half-marathoners. Fractional sodium excretion decreased significantly after running a M or HM. M, marathon; HM, half-marathon; baseline, 1–2 days before the run; peak, immediately after the run in the finishing area; recovery, after 2–7 days of recovery; **p < 0.01, ***p < 0.001.

Lactate increased significantly after running a M or HM (M_baseline vs. M_peak, p < 0.001; HM_baseline vs. HM_peak, p < 0.001) (Fig. 2a).

### Markers of red cell and muscle cell destruction

Free haemoglobin (free_hb) levels after running a HM were significantly above the baseline levels (HM_baseline vs. HM_peak, p < 0.001) but remained unchanged in M runners (Fig. 2b).

Creatine kinase (CK) and CK-MB increased in subjects taking part in a M immediately after the race (CK M_baseline vs. M_peak, p < 0.001, CK-MB M_baseline vs. M_peak, p = 0.007) and remained elevated after 2 to 7 days of recovery (Fig. 2c). These changes were not observed in HM runners.

### Inflammation

C-reactive protein (CRP) was significantly lower in marathoners at rest than in sedentary subjects (M_baseline vs. sedentary subjects, p = 0.004). There was no increase of CRP immediately after the race. CRP increased in marathoners after 2 to 7 days of recovery (M_peak vs. M_recovery, p < 0.001), but remained stable in HM runners (Fig. 2d).

### Electrolytes and renal function

Serum sodium (Na) increased significantly after running a M or HM (M_baseline vs. M_peak, p < 0.001; HM_baseline vs. HM_peak, p < 0.001) and both returned to baseline levels again after 2 to 7 days of recovery.

Fractional sodium excretion (FE_Na) and urine sodium (U_Na) declined significantly in M (FE_Na M_baseline vs. M_peak, p < 0.001, U_Na M_baseline vs. M_peak, p = 0.005) and HM runners (FE_Na HM_baseline vs. HM_peak, p = 0.004, U_Na HM_baseline vs. HM_peak, p = 0.022) immediately after the race (Fig. 2e).

Urine creatinine remained unchanged in M and HM runners.

Serum creatinine (Crea) increased significantly after running a M or HM (M_baseline vs. M_peak, p < 0.001; HM_baseline vs. HM_peak, p < 0.001) and returned to baseline levels again after 2 to 7 days of recovery.

### Liver function

Gamma-Glutamyl-Transferase (γGT) remained unchanged in all three cohorts.

### Serum concentrations of sRAGE, esRAGE, HMGB1 and AGE-CML

**Figure 3:**
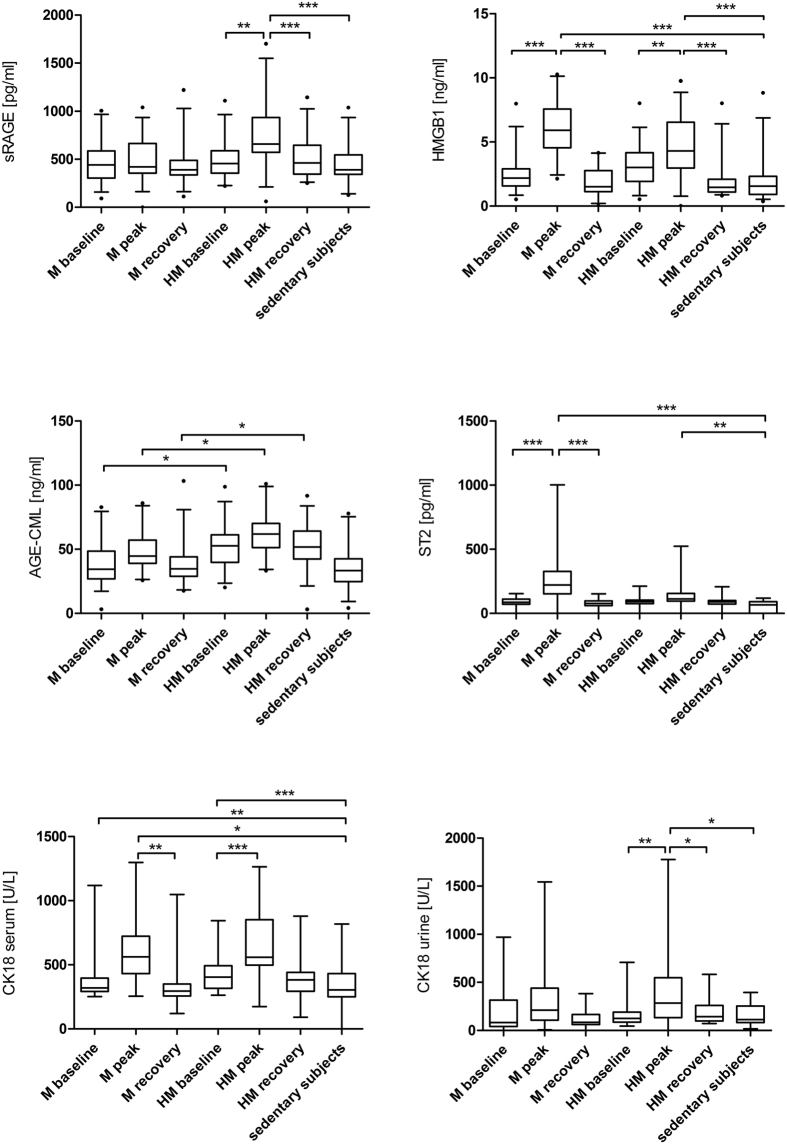
(**a**) Soluble RAGE in marathoners and half-marathoners. Soluble RAGE increased significantly after running a HM. Serum concentrations in M runners remained stable. M, marathon; HM, half-marathon; baseline, 1–2 days before the run; peak, immediately after the run in the finishing area; recovery, after 2–7 days of recovery; **p < 0.01, ***p < 0.001. (**b**) High mobility group box 1 in marathoners and half-marathoners. High mobility group box 1 increased significantly after running a M or HM. M, marathon; HM, half-marathon; baseline, 1–2 days before the run; peak, immediately after the run in the finishing area; recovery, after 2–7 days of recovery; **p < 0.01, ***p < 0.001. (**c**) Advanced Glycation Endproducts-Carboxymethyllysine in marathoners and half-marathoners. Advanced glycation endproducts-carboxymethyllysine was increased in HM runners at all three timepoints. M, marathon; HM, half-marathon; baseline, 1–2 days before the run; peak, immediately after the run in the finishing area; recovery, after 2–7 days of recovery. (**d**) Soluble ST2 in marathoners and half-marathoners. Soluble ST2 increased significantly after running a M or HM. ST2, suppressor of tumorgenicity 2; M, marathon; HM, half-marathon; baseline, 1–2 days before the run; peak, immediately after the run in the finishing area; recovery, after 2–7 days of recovery; **p < 0.01, ***p < 0.001. (**e**) Total cytokeratin 18 in marathoners and half-marathoners. Serum total CK18 concentrations increased significantly in amateur runners participating in M and HM. M, marathon; HM, half-marathon; baseline, 1–2 days before the run; peak, immediately after the run in the finishing area; recovery, after 2–7 days of recovery; *p < 0.05, **p < 0.01, ***p < 0.001. (**f**) Urine total cytokeratin 18 in marathoners and half-marathoners. Urine total CK18 concentrations increased significantly in amateur runners participating in HM. M, marathon; HM, half-marathon; baseline, 1–2 days before the run; peak, immediately after the run in the finishing area; recovery, after 2–7 days of recovery; *p < 0.05, **p < 0.01.

Baseline serum concentrations of sRAGE were equal in all three groups. We found a significant increase of sRAGE after running a HM (HM_baseline vs. HM_peak, p = 0.004), which returned to baseline after 2 to 7 days of recovery. SRAGE remained unchanged in M runners (Fig. 3a).

EsRAGE concentrations remained unchanged in M and HM runners (figure in supplements).

HMGB1 was significantly increased after M (M_baseline vs. M_peak, p < 0.001; M_peak vs. M_recovery, p < 0.001), and after HM (HM_baseline vs. HM_peak, p = 0.003; HM_peak vs. HM_recovery, p < 0.001; HM_peak vs. sedentary subjects, p < 0.001) (Fig. 3b).

AGE-CML was significantly increased in HM runners at all three time points when compared to M runners and sedentary controls (HM_baseline vs. M_baseline, p = 0.016; HM_baseline vs sedentary subjects, p = 0.003; HM_peak vs. M_peak, p < 0.006; HM_recovery vs. M_recovery, p = 0.015) (Fig. 3c).

### Serum concentrations of IL1-RA, ST2 and IL-33

Serum concentrations of IL1-RA were not affected by running a M or HM.

SST2 increased significantly in M runners following the race (peak) and returned to baseline levels after 2 to 7 days of recovery (M_baseline vs. M_peak, p < 0.001; M_peak vs. M_recovery, p < 0.001) (Fig. 3d).

Serum concentrations of IL33 were not affected by running a M or HM (figure in supplements).

### Serum and urinary concentrations as well as fractional excretion of CK 18

Baseline serum concentrations of serum total CK18 were significantly higher in M and HM runners at rest than in sedentary controls (M_baseline vs. control, p = 0.002; HM_baseline vs. control, p < 0.001). Serum total CK18 concentrations increased significantly in amateur runners participating in a M (M_peak vs. M_recovery, p = 0.001) and HM (HM_baseline vs. HM_peak, p < 0.001) (Fig. 3e). Urine total CK18 concentrations only increased significantly in HM runners after the race (HM_baseline vs. HM_peak, p = 0.008; HM_peak vs. HM_recovery, p = 0.049) (Fig. 3f).

Serum and urinary concentrations caspase-cleaved CK18 were not affected by running M or HM. The fractional total CK18 and the fractional caspase-cleaved CK18 excretion remained unchanged in M and HM runners.

## Discussion

To our knowledge, this is the first study investigating serum concentrations of sRAGE, esRAGE, HMGB1, AGEs, IL1-RA, ST2, IL33 and caspase-cleaved CK18 in recreational runners participating in a M or HM in parallel to routine laboratory examinations, lactate measurements and assessment of anthropometrical parameters. The combination of purely experimental measurements with routine clinical diagnostic testing was designed to find associations with heart, kidney, liver function, inflammation, dehydration and muscle damage.

Serum concentrations of acute phase proteins increase or decrease by at least 25% during acute or chronic inflammatory states associated with infection, trauma, infarction, inflammatory arthritis or other systemic autoimmune and inflammatory diseases, as well as various neoplasms^31^. This definition pertains to some of the measured cytokines in our study cohort including sRAGE (HM), HMGB1 (M + HM), ST2 (M + HM), as well as serum total CK18 (M + HM).

ELISA used for quantification of sRAGE concentrations do not separate between sRAGE-ligand complexes and free sRAGE^32^. Since there were no alterations measurable for esRAGE, only proteolytically cleaved sRAGE was released into the blood stream of M/HM athletes. An eight-week standardised aerobic high-amount-high-intensity training program led to a 61% increase in plasma esRAGE concentrations in sedentary subjects with low to intermediate risk for cardiovascular disease^33^. These findings are in contrast to the results of our study showing no difference in baseline esRAGE levels between sedentary subjects and participants who prepared for a M event. There was no standardized training for the participating amateur runners in our study. Preparation and training was individually assessed by a questionnaire.

The reasons for the selective serum sRAGE increase immediately after the HM race but not the M race in the present study remain elusive. We can only speculate that this is due to differences intrinsic to HM and M in non-professional athletes. Differences might stem from training habits (well-trained athletes have more muscle mitochondria) or exercise intensity. The intensity of exercising during long distance running determines deployment of different sources (glycogen stores and fat metabolism) for generation of energy. Resisting glycogen depletion and neurological fatigue can be obtained by training of economical running form and increasing the involved motor units to share in the work and reduce mitochondrial stress^34^. A combination of this fatigue resistance, running economy and minimization of homeostasis disturbance might be an explanation for stable parameters in M runners, which are influenced in HM runners. Resistance to oxidative stress induced by training and therefore an ability to tolerate higher amounts during a M event could be another explanation^35^.

HMGB1 can be actively secreted by immune cells under external stress or passively released by necrotic or apoptotic cells^36^. Decreased HMGB1 serum concentrations were detected in patients after myocardial infarction undergoing 6 months of exercise-based cardiac rehabilitation compared to patients only receiving instructions for lifestyle changes^37^. All of our study participants were in good health and none had been diagnosed to have cardiovascular diseases. Increased HMGB1 serum concentrations immediately after M and HM that returned to baseline levels during the recovery week may stem from immune cells, endothelial cells or cardiomyocytes. One might suspect burden to the heart for M runners since HMGB1 serum concentrations were paralleled by increases in CK-MB. Since in our HM cohort CK-MB serum concentrations were not increased and thus did not parallel the HMGB1 increase in HM runners one of the other sources may be more likely but remain elusive.

A source for AGEs was found in cooking methods as broiling, frying, roasting and boiling^38^. In middle-aged healthy women serum concentrations of AGE-CML adduct declined after a 12-week lifestyle modification^39^. A possible explanation for increased AGE-CML in HM runners could be poorer preparation for the running event, different eating habits and body composition. Relevant differences between the HM and M group and sedentary are significantly different BMI, body fat percentage and training days.

Rapid formation of AGE-CML adducts can be induced under conditions of oxidative stress, as evidenced by impaired AGE-CML generation in mice deficient in NADPH oxidase^40^. Reactive oxygen species and oxidative stress are induced by prolonged running^41^. Put together, this could be a possible alternative mechanism for increased AGE-CML serum concentrations at peak exercise.

SST2 increased significantly in patients with acute myocardial infarction and both sST2 and IL-33/sST2 ratio correlated with the 6-month prognosis^42^. ST2 is a marker of cardiac strain and fibrosis that gains in importance for risk stratification of patients with a broad spectrum of cardiovascular diseases. Guidelines from the 2013 American College of Cardiology and American Heart Association now recommend measurement of ST2 next to natriuretic peptides for further risk stratification in patients with acute or chronic ambulatory heart failure^12^. The myocardial stress in marathon running is undisputed. We detected increased sST2 serum concentrations in marathoners immediately after the run. This phenomenon might be similar to CK-MB increasing in asymptomatic marathoners arising from a non-cardiac or skeletal muscle source^43^.

Further, we investigated the anti-inflammatory cytokine IL1-RA, which was reported to be secreted during muscle contraction via IL6 activation^44^. Several studies observed an elevation of IL-1RA concentrations following endurance training or M. A 100-fold increase in IL-1RA concentrations was observed in plasma of 16 male runners after an M event^45^. After 6 hours of endurance running an increase of IL1-RA plasma concentrations could be demonstrated in 19 well-trained athletes^46^. In 14 male triathletes participating in a duathlon race neutrophil reactive oxygen species production and migratory neutrophil count *ex vivo* were both correlated with IL-1RA plasma concentrations^47^. In contrast to these results there were no elevations in IL-1RA serum concentrations in our study cohort. We can only speculate on the rise of IL-1RA in other studies: in all other studies plasma (and not serum) was investigated. None of the studies investigated female runners. Our study investigated non-professional runners. More intense endurance training and performance directed towards winning the race (e.g. sprinting towards the finish line) in more professional athletes may yield different cytokine concentrations.

The cellular changes associated with the sports medical phenomenon of the “open window effect” were reproduced as expected from previous reports: increased numbers of leucocytes, neutrophils and platelets with a concomitant decrease of lymphocytes and monocytes in the blood stream^48^. White blood cells and platelets are known to be increased after running a M^49^. Following strenuous exercise and excessive overtraining lymphocytes are likely to fall below pre-exercise levels leading to a higher vulnerability towards infectious diseases^50^. Interestingly, monocytes decreased significantly only after running a HM, but not after M. We did not detect a higher number of viral respiratory diseases associated with the open window effect in our study cohort.

Running a M was reported to increase biomarkers that are relevant to the diagnosis of liver injury such as Gamma-Glutamyl-Transferase (γGT), Aspartate transaminase (AST), lactate dehydrogenase (LDH) and bilirubin, which arise from muscle injury, cardiac stress or hemolysis. In our study cohort γGT remained unchanged in all subjects and through all three time points.

CK was demonstrated to peak after 24 hours following a M event and not to return to baseline levels for over a week, indicating rhabdomyolysis and muscle damage^51^,^52^. We made the same experience in our M and HM runners where CK and CK-MB serum concentrations were still significantly increased after 2 to 7 days of recovery.

There is no published literature investigating free hemoglobin in M and HM runners. The reasons for increased free hemoglobin concentrations in half-marathoners, but not in marathoners remain elusive.

The isolated increase of total CK18 and unchanged levels of caspase-cleaved CK18 indicate the prevalence of necrosis rather than apoptosis. This could arise either from necrosis of epithelial and endothelial cells or from increased disturbance of immune functions and increased release of pro-inflammatory cytokines^53^.

In a cross-sectional study investigating 147 recreational male HM and 126 recreational male M runners differences in anthropometry and training characteristics were shown. HM runners had higher amounts of total skinfold thickness, a higher body fat percentage and skeletal muscle mass than M runners. Regarding training characteristics, HM participants were running less kilometre and fewer hours per week, completed fewer and shorter trainings sessions and were running for fewer years^48^. In our study cohort we had the same experience that HM runners were running significantly less kilometre and fewer hours per week than M runners. BMI, abdominal girth and body fat were significantly lower in marathoners than in half-marathoners. In the study mentioned above only men were included. Women were more likely to attend the HM than the M explaining differences in body fat measurements between M and HM in our study cohort.

In eight endurance-trained runners CRP did not change during a M or at the finish line. An increase was observed 24 and 48 hours after finishing the race. In 15 professional athletes a decrease in neutrophil phagocytic activity, increase in apoptosis and necrosis of lymphocytes and decreased production of IL-2, TNF-α, IL-1β and IL-10 by lymphocytes was observed. While IL-6 and IL1-RA plasma concentrations increased, plasma CRP concentrations remained unchanged immediately after the race^54^. In our study cohort CRP concentrations did not change immediately after the race but were significantly elevated during the recovery period in M runners. There is evidence that intensive regular exercise could have a systemic anti-inflammatory effect, as CRP serum levels decreased following nine months of endurance training in 12 marathon runners^55^. In contrast, we observed significantly lower resting CRP concentrations in non-professional M but not in HM runners.

CRP is an acute phase protein used in clinical routine to detect ongoing inflammation or infection. It is not surprising, that CRP concentrations are unchanged in our population after running a M or HM since CRP synthesis needs about 6 to 8 hours to start and peak concentrations are reached between 36 to 50 hours after a stimulus^56^.

Plasma volume changes due to dehydration in M runners are common and result from heavy sweating and, to a smaller extent from urination and respiration. Sweat rates are determined by environmental conditions and acclimatization, trainings status, exercise intensity, clothing and genetics^57^. Plasma volumes in M runners were reported to be reduced from pre-marathon volumes by 12.1%^58^. Electrolyte changes appear uncommon in M runners^59^. In our study cohort we found a significant plasma volume increase in marathoners and significant plasma volume decrease in half-marathoners. Fluid intake of 200 to 300 ml every 10 to 20 minutes is recommended to maintain hydration^57^,^60^. We can only speculate about the significant volume contraction in HM runners that was not observed in M runners. Marathoners in this study trained more frequently and longer distances. We attribute the missing plasma volume contraction in the non-professional M cohort on more experience in adequate fluid intake during the race. Serum sodium concentrations were significantly increased above the reference range in M and HM runners immediately after the race.

Severe dehydration, commonly observed in marathoners, can lead to pre-renal azotemia and subsequently to acute kidney injury^59^. In a prospective cohort study including 25 subjects 40% were identified to develop Stage 1 acute kidney injury, defined as a 50% rise in serum creatinine after participating in a M^11^. In our population we found 10/34 (29%) marathoners meeting the definition of AKI stage 1 and 2/34 (6%) AKI stage 2 according to the definition of the Acute Kidney Injury Network^61^. In athletes running a HM 11/36 (31%) met the criteria for AKI stage 1 and 5/36 (14%) for stage 2. There was no instruction to runners on fluid or food intake during the competition. Assessment of AKI stage is not necessarily based on urine volume. AKI stage was based on serum creatinine corrected for plasma changes^61^. After two to seven days of recovery creatinine concentrations returned to baseline levels without treatment or intervention. This effect was described before^62^. The long-term effects of these acute creatinine elevations remain unknown.

Blood lactate concentrations at rest are about 0.5–1.5 mmol/L and can increase up to 20 mmol/L or even more during high intensity exercise involving a maximum effort over 5 to 10 minutes. M running is a long lasting exercise at slower running speed and therefore lactate concentrations immediately after running a M do not exceed 3 mmol/L^63^. In our runners participating in a M or HM lactate concentrations increased significantly within the reported range.

## Conclusion

The present study brought new insights into the expression of cytokines before, immediately after and during the recovery week of M and HM events. We have gained valuable insight into the potential use of biomarkers that are currently evaluated as markers of disease activity. Further studies are warranted to define the role of the RAGE axis, ST2 and CRP in sports physiology.

## Material and methods

### Setting

In this prospective study male and female volunteers who participated in the Vienna City Marathon running the full M (42.195 km) or the HM distance (21.0975 km) were included. Subjects were studied three times, before the event at the time of registration (an event called the Vienna Sports World, further referred to as baseline), immediately after the event (peak) at the finish area, and two to seven days after the competition (recovery). At baseline, the study participants were asked to complete a standardized questionnaire to gather information on physical characteristics such as exercise during the last year and personal information such as smoking, drinking or eating habits. Body composition was assessed by Omron BF306 (Omron Healthcare Europe B.V., Netherlands) and a body fat calibre (Accu-Measure Fitness 3000 Body Fat caliper, AccuFitness, LLC PO Box 4411 Greenwood Village, CO 80155-4411). The study was conducted at the research laboratory of the department of thoracic surgery in collaboration with trauma and thoracic surgeons, sports and laboratory physicians, Red Cross paramedics and medical students at the Vienna City Marathon, held on April 15^th^, 2012.

### Study population

At the Vienna Sports World, a fair organized by the Vienna City Marathon team one or two days prior to the marathon, we enrolled 54 M and 59 HM runners. Twenty M and 23 HM runners did not finish the race or did not complete all tests and were therefore excluded from the study analysis. Thirty-four M, 36 HM runners and 30 non-running sedentary controls met the inclusion criteria for the study and completed follow-up.

None of the participants received anti-inflammatory or immunosuppressant therapy, reported current or recent infections, illness or chronic diseases or had elevated inflammatory parameters, as assessed by pre-competition CRP and leukocyte levels.

Ethical approval was obtained from the institutional review board (EK 1034/2012) of the Medical University Vienna. All tests were performed in accordance with the Declaration of Helsinki and the guidelines for good scientific practice of the Medical University Vienna. All subjects participating in this study gave written informed consent.

### Laboratory procedures

Blood samples were taken at baseline, peak and recovery. Blood was collected from the antecubital vein and was collected in EDTA (Greiner Bio-One, REF: 455036) and serum gel tubes (Greiner bio-one, REF: 455071). EDTA tubes were used for blood count analysis using a haematology analyser (Sysmex KX-21N) and for investigations of routine parameters in the clinical institute of laboratory medicine.

Serum was separated by centrifugation (15 minutes at RCF 2845 × g) and used for quantification of the indicated proteins.

For lactate measurements, venous blood was collected in end-to-end capillaries, stored in hemolysis solution (Glucocapil, Hitado Diagnostics, Delecke, Germany) and analyzed using Super GL ambulance (Ruhrtal Labor Technik, Möhnesee, Germany).

Urine samples were collected in urine cups used in clinical routine.

All samples were stored at −80 °C within 2 hours after the event until further processing.

### Detection of proteins in serum and urine

Concentrations of the indicated proteins in serum and urine were quantified by enzyme-linked immunosorbent assays (ELISA). All ELISA assays were commercially available and performed according to the manufacturers’ instructions. The employed immunoassays were purchased as follows: sRAGE (RnD Systems, USA), esRAGE (B-Bridge International Inc., USA), HMGB1 (IBL International GmbH, Germany), Advanced glycation endproducts carboxymethyllysine (AGE-CML) (MicroCoat Biotechnologie GmbH, Germany), ST2 (RnD Systems), IL1-RA (RnD Systems) and IL33 (RnD Systems). For quantitative determination of total and caspase cleaved CK-18 in serum and urine we used M30 (measuring ccCK18) and M65 ELISA (measuring total CK18) kits (Peviva AB, Sweden). The formula used for the calculation of the fractional CK 18 excretion was: ((urine CK-18 × serum creatinine)/(serum CK-18 × urine creatinine)) × 100^29^. Researchers performing the laboratory work and data analyses were blinded to the study groups associated with each sample.

### Correction for changes in plasma volume

All measurements were corrected for changes in plasma volume. Plasma volume changes due to dehydration in marathoners is common and is a result of heavy sweating and in a small extent a loss of body water with urine and respiratory losses^57^. These changes were calculated as described by Dill and Costill and in a more recent publication in marathoners^64^,^65^.

### Statistical methods

Statistical analysis of data was performed using SPSS software (version 22; IBM SPSS Inc., IL, USA). Kruskal-Wallis rank test was used to evaluate non-normal distributions. Unpaired student’s t tests and one-way ANOVA were used to compare means of two or more than two independent groups with normal (Gaussian) distributions, respectively. The type of used test is indicated in the table and/or the results section. Post hoc comparisons were computed with Tukey’s-B and Bonferroni correction. All data were reported as mean ± standard error of the mean in the text of the results section and more detailed as mean (median) ± standard deviation (standard error of the mean) in tables. Chi-square test for independence was used for analysis of frequencies or distributions of nominal values in two or more groups. The probability of making a type I error was set at α value of 0.05. The null hypothesis was rejected if the p-value was less than α. Two-tailed p-values were employed. GraphPad Prism 6 (GraphPad Software Inc., California, USA) was used to create figures.

## Additional Information

**How to cite this article**: Bekos, C. *et al*. Non-professional marathon running: RAGE axis and ST2 family changes in relation to open-window effect, inflammation and renal function. *Sci. Rep.* **6**, 32315; doi: 10.1038/srep32315 (2016).

## Supplementary Material

Supplementary Information

## Figures and Tables

**Table 1:** Basic characteristics of 34 marathoners, 36 half-marathoners and 30 sedentary subjects.

	M	HM	Sedentary subjects	p-value
Age (years)	36.83 (36.50) ± 7.56 (1.28)	36.89 (37.00) ± 7.90 (1.34)	32.50 (29.00) ± 9.72 (1.77)	0.063^a^
F:M (quantity)	10:24	17:19	13:17	0.430^a^
BMI (kg/m2)	22.29 (22.51) ± 2.16 (0.38)	24.05 (23.47) ± 3.56 (0.60)	24.22 (22.99) ± 4.66 (0.92)	0.046^a^
weight (kg)	69.35 (71.00) ± 10.04 (1.75)	73.55 (71.85) ± 15.64 (2.64)	74.48 (73.00) ± 14.28 (2.65)	0.223^a^
abdominal girth (cm)	77.41 (78.00) ± 10.69 (1.86)	84.19 (82.50) ± 11.78 (1.96)	85.72 (85.00) ± 16.98 (3.10)	0.025^a^
hip size (cm)	89.91 (91.00) ± 7.20 (1.25)	94.04 (93.00) ± 8.74 (1.46)	96.48 (97.50) ± 15.68 (2.86)	0.060^a^
body fat percentage (%)	18.45 (17.40) ± 7.11 (1.24)	25.31 (24.45) ± 6.18 (1.03)	22.58 (21.45) ± 9.20 (1.68)	0.001^a^
Smoker:Nonsmoker:Neversmoker (quantity)	01:08:25	02:06:28	03:04:23	0.502^b^
Pack years (years)	1.7 (0) ± 4.59 (0.85)	3.23 (0) ± 7.24 (1.28)	3.32 (0) ± 9.82 (1.86)	0.627^a^

All results are reported as mean (median) ± standard deviation (standard error mean). f:m ratio, female:male ratio; BMI, body mass index; M, marathon; HM, half-marathon.

^a^One-way ANOVA.

^b^Pearson’s *χ*^*2*^ test for independence.

**Table 2:** Running and training history of 34 marathoners and 36 half-marathoners.

	M	HM	p-value
marathon time (minutes)	226.79 (230.00) ± 31.09 (5.33)	117.00 (112.00) ± 15.92 (2.77)	<0.001^c^
km per week	61.85 (60.00) ± 20.58 (3.96)	35.10 (35.00) ± 14.45 (2.64)	<0.001^c^
training days per week	4.47 (4.00) ± 1.05 (0.19)	3.53 (3.00) ± 1.21 (0.20)	0.001^c^

^c^Unpaired student’s t-test.

All results are reported as mean (median) ± standard deviation (standard error mean). Km, kilometre; M, marathon; HM, half-marathon.
