# Supplementary material for: Non-professional marathon running: RAGE axis and ST2 family changes in relation to open-window effect, inflammation and renal function
Source: Sci Rep. 2016 Sep 22;6:32315. doi: 10.1038/srep32315 (PMC5032027; doi:10.1038/srep32315)
Supplement: Supplementary Information [file srep32315-s1.pdf]

Non-professional marathon running: RAGE axis and ST2 family changes in relation to open-window effect, inflammation and renal function

Christine Bekos[1,2], Matthias Zimmermann[1], Lukas Unger[1], Stefan Janik[1], Philipp Hacker[1], Andreas Mitterbauer[1], Michael Koller[3], Robert Fritz[3], Mario Kessler[4], Stefanie Nickl[5], Jessica Didcock[1], Patrick Altmann[1], Thomas Haider[1,6], Georg Roth[7], Walter Klepetko[8], Hendrik Jan Ankersmit[1,8] and Bernhard Moser[1,8*]

[1] Christian Doppler Laboratory for Cardiac and Thoracic Diagnosis and Regeneration, Medical University Vienna, Austria

[2] Department. of Obstetrics and Gynaecology, Division of Gynaecology and Gynecological Oncology, Medical University Vienna, Austria

[3] Sportordination, Alserstraße 27/1/6, Vienna, Austria

[4] Austrian Red Cross, Nottendorfer Gasse 21, 1030 Wien, Austria

[5] Department of Surgery, Division of Plastic and Reconstructive Surgery, Medical University Vienna, Austria

[6] Department of Trauma Surgery, Medical University Vienna, Austria

[7] Department of Anesthesia, Critical Care and Pain Medicine, Medical University Vienna, Austria

[8] Department of Surgery, Division of Thoracic Surgery, Medical University Vienna, Austria

*Corresponding author
Bernhard Moser, MD, Assoc. Prof., FEBTS
Department of Thoracic Surgery, Division of Surgery, Medical University Vienna
Währinger Gürtel 18-20, 1090 Vienna, Austria
FAX: +43 1 40400 69770
Tel: +43 1 40400 67770
E-mail: bernhard.moser@meduniwien.ac.at

Supplemental material

Table. Red and white blood count analysis in 34 marathoners, 36 half-marathoners and 30 sedentary controls before the run (baseline), immediately after (peak) and after 2 to 7 days of recovery (recovery).

	M baseline	M peak	M recovery	HM baseline	HM peak	HM recovery	Sedentary subjects	p-value
**WBC**	6.51 (6.05) ± 1.71 (0.29)	17.08 (16.16) ± 4.10 (0.70)	6.88 (6.55) ± 1.79 (0.31)	6.44 (6.65) ± 1.40 (0.23)	12.20 (11.52) ± 3.68 (0.62)	6.53 (6.65) ± 1.40 (0.23)	6.71 (6.15) ± 1.88 (0.34)	<0.001[a]
**RBC**	4.96 (4.94) ± 0.35 (0.06)	5.36 (5.33) ± 0.49 (0.08)	4.81 (4.83) ± 0.37 (0.06)	4.82 (4.81) ± 0.42 (0.07)	5.18 (5.33) ± 0.59 (0.10)	4.61 (4.60) ± 0.42 (0.07)	4.96 (4.98) ± 0.59 (0.11)	<0.001[a]
**HGB**	14.62 (14.75) ± 1.20 (0.21)	15.50 (16.00) ± 1.41 (0.24)	14.08 (14.20) ± 1.37 (0.24)	14.08 (14.10) ± 1.10 (0.18)	15.21 (14.93) ± 1.47 (0.25)	13.45 (13.45) ± 1.10 (0.18)	14.37 (14.40) ± 1.53 (0.28)	<0.001[a]
**HCT**	44.58 (44.20) ± 2.77 (0.48)	44.42 (44.40) ± 3.10 (0.53)	42.87 (43.15) ± 3.35 (0.58)	43.10 (43.00) ± 2.88 (0.48)	43.39 (43.10) ± 3.31 (0.56)	41.14 (41.65) ± 2.86 (0.48)	43.90 (43.60) ± 4.46 (0.81)	<0.001[a]
**MCV**	90.06 (90.35) ± 4.68 (0.80)	95.84 (97.07) ± 5.19 (0.89)	89.25 (90.15) ± 5.55 (0.95)	89.61 (89.60) ± 3.75 (0.62)	95.91 (96.00) ± 4.14 (0.70)	89.49 (89.65) ± 3.86 (0.64)	88.76 (89.30) ± 4.95 (0.90)	<0.001[a]
**MCH**	29.54 (29.90) ± 1.98 (0.34)	31.53 (32.00) ± 2.18 (0.37)	29.30 (29.75) ± 2.35 (0.40)	29.25 (29.05) ± 1.37 (0.23)	31.39 (30.93) ± 1.64 (0.28)	29.23 (29.05) ± 1.53 (0.26)	29.06 (29.15) ± 2.09 (0.38)	<0.001[a]
**MCHC**	32.78 (32.95) ± 1.07 (0.18)	35.01 (35.20) ± 1.16 (0.20)	32.80 (33.00) ± 1.06 (0.18)	32.64 (32.55) ± 0.71 (0.12)	34.93 (35.20) ± 0.83 (0.14)	32.68 (32.65) ± 0.86 (0.14)	32.73 (32.85) ± 1.14 (0.21)	<0.001[a]
**PLT**	260.21 (252.50) ± 62.29 (10.68)	334.05 (328.00) ± 68.54 (11.75)	249.88 (250.00) ± 49.076 (8.42)	246.61 (242.00) ± 50.06 (8.34)	316.50 (301.87) ± 76.63 (12.95)	242.75 (234.50) ± 47.42 (7.90)	230.77 (231.00) ± 52.45 (9.58)	<0.001[a]
**LYM**	30.28 (29.85) ± 7.26 (1.25)	11.34 (10.67) ± 4.44 (0.76)	30.86 (30.10) ± 8.14 (1.40)	34.42 (31.35) ± 11.58 (1.93)	18.71 (17.92) ± 6.76 (1.14)	32.61 (31.65) ± 7.21 (1.20)	32.60 (31.10) ± 8.50 (1.55)	<0.001[a]
**MXD**	8.28 (7.60) ± 3.30 (0.59)	7.48 (7.25) ± 2.61 (0.45)	7.99 (6.50) ± 3.30 (0.59)	9.49 (9.50) ± 2.27 (0.39)	6.77 (6.61) ± 2.29 (0.38)	8.81 (8.45) ± 2.21 (0.37)	8.51 (8.00) ± 2.80 (0.56)	0.002[a]
**NEUT**	61.96 (62.70) ± 8.32 (1.49)	85.08 (88.43) ± 15.91 (2.73)	61.10 (62.20) ± 9.08 (1.63)	58.12 (59.60) ± 7.76 (1.33)	81.19 (81.49) ± 6.96 (1.18)	58.57 (59.15) ± 7.70 (1.28)	59.14 (59.40) ± 8.84 (1.77)	<0.001[a]
**RDW_SD**	45.34 (44.85) ± 2.71 (0.47)	47.43 (46.83) ± 3.15 (0.54)	44.73 (44.20) ± 2.79 (0.48)	44.43 (44.85) ± 2.31 (0.38)	47.17 (47.57) ± 2.81 (0.48)	44.05 (44.30) ± 2.34 (0.40)	44.11 (43.50) ± 2.31 (0.43)	<0.001[a]
**RDW_CV**	13.25 (13.00) ± 1.23 (0.21)	13.90 (13.55) ± 1.33 (0.23)	13.26 (12.90) ± 1.53 (0.26)	12.94 (13.00) ± 0.57 (0.09)	13.75 (13.76) ± 0.68 (0.11)	12.91 (12.90) ± 0.56 (0.10)	13.62 (12.95) ± 3.55 (0.65)	0.062[a]
**PDW**	13.41 (13.00) ± 1.91 (0.33)	14.79 (14.61) ± 2.16 (0.37)	12.49 (12.40) ± 1.85 (0.32)	13.70 (13.90) ± 1.63 (0.27)	14.95 (15.15) ± 1.90 (0.32)	12.75 (12.60) ± 1.81 (0.31)	13.21 (13.10) ± 2.14 (0.40)	<0.001[a]
**MPV**	10.24 (10.10) ± 0.89 (0.15)	11.16 (11.25) ± 0.96 (0.17)	9.80 (9.70) ± 0.91 (0.16)	10.42 (10.60) ± 0.88 (0.15)	11.24 (11.41) ± 0.85 (0.14)	9.90 (9.90) ± 0.90 (0.15)	10.07 (10.10) ± 1.04 (0.19)	<0.001[a]
**P_LCR**	27.64 (26.45) ± 6.97 (1.20)	31.36 (31.79) ± 7.58 (1.30)	24.13 (23.15) ± 6.87 (1.18)	28.94 (30.65) ± 6.60 (1.10)	31.83 (33.07) ± 6.70 (1.13)	25.22 (25.20) ± 6.90 (1.17)	26.28 (26.30) ± 7.90 (1.47)	<0.001[a]

All results are reported as mean (median) ± standard deviation (standard error mean).

M, marathon; HM, half-marathon; baseline, 1-2 days before the run; peak, immediately after the run in the finishing area; recovery, after 2-7 days of recovery; WBC, white blood cells; RBC, red blood cells; HGB, hemoglobin; HCT, hematocrit; MCV, mean corpuscular volume; MCH, mean corpuscular hemoglobin; MCHC, mean corpuscular hemoglobin concentration; PLT, platelets; LYM, lymphocytes; MXD, monocytes; NEUT, neutrophils; RDW_SD, red cell distribution width reported statistically as coefficient of variation; RDW_CV, red cell distribution width reported statistically as standard

deviation; PDW, platelet distribution width; MPV, mean platelet volume; P_LCR, platelet larger cell ratio.

Table. Laboratory analysis in 34 marathoners, 36 half marathoners and 30 sedentary controls before the run (baseline), immediately after (peak) and after 2 to 7 days of recovery (recovery).

	M baseline	M peak	M recovery	HM baseline	HM peak	HM recovery	Sedentary subjects	p-value
**Lactate**	0.88 (0.72) ± 0.41 (0.08)	3.02 (2.98) ± 1.48 (0.26)		0.88 (0.74) ± 0.38 (0.07)	3.09 (2.87) ± 1.55 (0.27)		1.15 (1.03) ± 0.59 (0.15)	<0.001[a]
**Sodium**	136.21 (137.00) ± 4.12 (0.71)	149.11 (150.93) ± 6.11 (1.05)	135.69 (138.00) ± 11.04 (2.05)	134.11 (139.00) ± 13.74 (2.29)	150.01 (152.53) ± 8.33 (1.39)	139.93 (140.00) ± 3.98 (0.70)	135.62 (136.00) ± 7.26 (1.35)	<0.001[a]
**Creatinine**	0.85 (0.86) ± 0.11 (0.02)	1.26 (1.14) ± 0.30 (0.05)	0.81 (0.84) ± 0.16 (0.03)	0.79 (0.79) ± 0.17 (0.03)	1.22 (1.22) ± 0.21 (0.03)	0.87 (0.89) ± 0.13 (0.02)	0.82 (0.81) ± 0.15 (0.42)	<0.001[a]
**Free Hb**	4.54 (2.67) ± 5.02 (0.86)	5.45 (4.60) ± 4.26 (0.73)	4.17 (2.83) ± 3.58 (0.68)	2.69 (2.34) ± 1.73 (0.29)	11.75 (6.33) ± 13.93 (2.32)	6.15 (4.10) ± 5.90 (1.04)	3.33 (2.62) ± 2.28 (0.42)	<0.001[a]
**γ-GT**	18.24 (16.00) ± 9.86 (1.69)	20.74 (18.13) ± 10.24 (1.76)	16.79 (15.00) ± 7.85 (1.46)	20.22 (13.00) ± 18.37 (3.06)	22.67 (16.00) ± 19.51 (3.25)	21.84 (14.50) ± 19.86 (3.51)	18.90 (17.00) ± 8.29 (1.54)	0.696[a]
**CK**	157.44 (141.00) ± 68.53 (11.75)	475.29 (437.33) ± 209.39 (35.91)	297.97 (203.00) ± 262.83 (48.81)	131.92 (115.00) ± 70.53 (11.76)	226.28 (220.27) ± 94.85 (15.81)	268.47 (232.50) ± 166.82 (29.49)	158.79 (129.00) ± 139.15 (25.84)	<0.001[a]
**CK-MB**	(N=10) 16.59 (14.40) ± 11.19 (3.54)	29.49 (26.29) ± 12.87 (2.21)	17.39 (15.90) ± 7.39 (1.70)	10.67 (10.50) ± 2.89 (1.09)	19.76 (16.48) ± 9.33 (2.20)	14.01 (13.30) ± 4.37 (1.03)	16.95 (15.55) ± 7.34 (3.00)	<0.001[a]
**FE_Na**	0.79 (0.72) ± 0.42 (0.07)	0.34 (0.22) ± 0.35 (0.06)	0.82 (0.75) ± 0.39 (0.07)	0.77 (0.68) ± 0.43 (0.08)	0.40 (0.34) ± 0.29 (0.05)	0.76 (0.65) ± 0.52 (0.10)	0.76 (0.69) ± 0.38 (0.08)	<0.001[a]
**Na_Urine**	87.94 (81.50) ± 58.89 (10.41)	36.22 (27.50) ± 28.81 (5.09)	103.19 (87.50) ± 66.99 (11.84)	93.97 (82.00) ± 61.68 (10.74)	49.79 (39.00) ± 35.82 (6.14)	95.97 (93.00) ± 57.64 (10.35)	120.88 (122.50) ± 69.06 (13.54)	<0.001[a]
**Crea_Urine**	90.88 (84.15) ± 67.87 (12.00)	154.81 (105.05) ± 135.54 (23.96)	96.47 (81.30) ± 75.35 (13.32)	84.87 (68.40) ± 61.54 (10.71)	156.83 (110.00) ± 142.13 (24.38)	96.35 (92.05) ± 66.42 (12.13)	110.14 (100.20) ± 80.26 (16.05)	0.005[a]
**CRP**	0.04 (0.00) ± 0.07 (0.01)	0.03 (0.00) ± 0.06 (0.01)	0.24 (0.13) ± 0.31 (0.06)	0.09 (0.05) ± 0.11 (0.02)	0.10 (0.06) ± 0.12 (0.02)	0.14 (0.11) ± 0.14 (0.02)	0.21 (0.11) ± 0.30 (0.06)	<0.001[a]

All results are reported as mean (median) ± standard deviation (standard error mean).

M, marathon; HM, half-marathon; baseline, 1-2 days before the run; peak, immediately after the run in the finishing area; recovery, after 2-7 days of recovery; free Hb, free haemoglobin; γ-GT, gamma-glutamyl-transferase; CK, creatine kinase; CK-MB, creatine kinase muscle-brain type; FE_Na, fractional sodium excretion; Na_Urine, urine sodium; Crea_urine, creatinine urine; CRP, C-reactive protein.

[a] one-way ANOVA

Table. Serum concentrations of sRAGE, esRAGE, HMGB1, AGE-CML, IL1-RA, ST2, IL33, ccCK18 serum and urine, CK18 serum and urine, fract ccCK18, fract CK18 in 34 marathoners, 36 half-marathoners and 30 sedentary controls before the run (baseline), immediately after (peak) and after 2 to 7 days of recovery (recovery).

	M baseline	M peak	M recovery	HM baseline	HM peak	HM recovery	Sedentary subjects	p-value
**sRAGE (pg/ml)**	469.87 (441.68) ± 236.92 (40.63)	519.74 (424.90) ± 229.25 (39.90)	445.78 (391.18) ± 219.56 (38.81)	532.36 (478.42) ± 210.97 (37.89)	761.46 (658.20) ± 344.71 (62.94)	456.10 (411.89) ± 168.32 (31.26)	452.68 (390.83) ± 204.75 (37.38)	<0.001[a]
**esRAGE (ng/ml)**	0.35 (0.31) ± 0.25 (0.04)	0.47 (0.39) ± 0.32 (0.05)	0.31 (0.29) ± 0.16 (0.03)	0.39 (0.34) ± 0.22 (0.04)	0.50 (0.48) ± 0.26 (0.04)	0.42 (0.38) ± 0.23 (0.04)	0.34 (0.32) ± 0.14 (0.03)	0.004[a]
**HMGB1 (ng/ml)**	2.58 (2.18) ± 1.58 (0.27)	6.02 (5.91) ± 2.18 (0.43)	1.86 (1.50) ± 1.08 (0.19)	3.13 (3.01) ± 1.63 (0.27)	4.78 (4.32) ± 2.10 (0.38)	2.02 (1.49) ± 1.55 (0.27)	2.00 (1.54) ± 1.73 (0.32)	<0.001[a]
**AGE-CML (ng/ml)**	38.42 (34.46) ± 16.18 (2.78)	48.04 (44.70) ± 16.01 (2.75)	38.07 (34.70) ± 16.65 (2.94)	51.98 (52.73) ± 17.17 (2.86)	62.63 (61.75) ± 16.95 (2.82)	52.22 (51.75) ± 16.96 (2.95)	35.63 (33. 37) ± 16.16 (3.05)	<0.001[a]
**IL1-RA (pg/ml)**	681.66 (87.15) ± 1012.61 (173.66)	926.91 (187.29) ± 1112.84 (190.85)	563.61 (0.00) ± 946.62 (162.34)	847.93 (150.57) ± 1039.88 (173.31)	951.57 (269.70) ± 1090.45 (181.74)	843.29 (181.76) ± 1047.72 (177.10)	679.11 (105.20) ± 863.07 (166.10)	0.668[a]
**ST2 (pg/ml)**	79.31 (85.07) ± 46.54 (7.98)	292.28 (230.88) ± 222.51 (38.73)	73.35 (77.21) ± 39.30 (6.95)	84.64 (90.26) ± 44.38 (7.50)	152.51 (115.01)) ± 95.08 (16.07)	89.88 (89.80) ± 36.90 (6.52)	57.23 (66.89) ± 42.73 (8.08)	<0.001[a]
**IL33 (pg/ml)**	337.45 (58.28) ± 517.23 (90.04)	357.55 (91.93) ± 493.16 (85.85)	283.95 (66.30) ± 461.80 (85.75)	284.50 (64.02) ± 412.50 (68.75)	308.25 (72.39) ± 463.82 (77.30)	283.77 (71.52) ± 406.30 (68.68)	235.89 (44.81) ± 392.25 (72.84)	0.960[a]
**ccCK18 Serum (U/l)**	112.76 (106.03) ± 42.77 (7.81)	121.80 (105.22) ± 52.84 (9.65)	116.70 (107.86) ± 44.99 (8.22)	130.12 (114.21) ± 55.17 (10.43)	146.28 (122.12) ± 68.39 (12.92)	137.56 (117.12) ± 85.70 (17.14)	152.80 (103.25) ± 156.42 (34.13)	0.403[a]
**ccCK18 Urine (U/l)**	72.25 (59.16) ± 50.91 (9.80)	118.47 (80.23) ± 120.15 (21.94)	89.08 (56.91) ± 110.24 (20.47)	47.76 (57.75) ± 31.07 (6.34)	120.27 (87.94) ± 149.38 (29.88)	41.62 (48.20) ± 41.69 (9.10)	57.43 (62.34) ± 32.69 (7.31)	0.008[a]
**CK18 Serum (U/l)**	376.99 (319.46) ± 164.44 (28.63)	611.14 (562.28) ± 268.70 (47.50)	325.93 (294.42) ± 157.47 (28.28)	433.05 (403.21) ± 149.74 (28.82)	651.65 (557.68) ± 273.11 (53.56)	389.24 (381.52) ± 153.19 (31.94)	342.61 (304.18) ± 181.51 (33.14)	<0.001[a]
**CK18 Urine (U/l)**	192.22 (82.30) ± 235.68 (42.33)	361.32 (210.82) ± 413.24 (77.29)	117.14 (83.36) ± 95.10 (17.66)	158.52 (123.97) ± 133.82 (26.76)	437.24 (284.15) ± 435.35 (95.00)	187.21 (143.05) ± 127.77 (28.57)	163.59 (112.20) ± 114.66 (22.93)	<0.001[a]
**Fract_ccCK 18**	1.10 (0.62) ± 1.01 (0.19)	1.24 (0.89) ± 1.23 (0.22)	1.51 (0.58) ± 2.20 (0.41)	0.66 (0.35) ± 0.67 (0.14)	1.46 (1.01) ± 1.38 (0.27)	0.57 (0.26) ± 0.81 (0.19)	0.90 (0.42) ± 1.27 (0.28)	0.008[a]
**Fract_CK18**	0.73 (0.33) ± 1.24 (0.22)	0.64 (0.35) ± 0.73 (0.14)	0.52 (0.27) ± 0.53 (0.10)	0.44 (0.38) ± 0.39 (0.08)	1.24 (0.63) ± 1.56 (0.33)	0.52 (0.46) ± 0.44 (0.11)	0.46 (0.25) ± 0.52 (0.11)	0.072[a]

All results are reported as mean (median) ± standard deviation (standard error mean). M, marathon; HM, half-marathon; baseline, 1-2 days before the run; peak, immediately after the run in the finishing area; recovery, after 2-7 days of recovery; sRAGE, soluble RAGE; esRAGE, endogenous secretory RAGE; HMGB1, high mobility group box1; AGE-CML, advanced glycation endproducts-carboxmethyllysine; IL1-RA, interleukin 1 receptor antagonist; ST2, suppressor of tumorgenicity 2; IL33, interleukin 33; ccCK18,, caspase-cleaved cytokeratin 18; CK18, total cytokeratin 18; Fract_ccCK18, fractional caspase-cleaved cytokeratin 18; Fract_CK18, fractional cytokeratin 18.

[a] one-way ANOVA

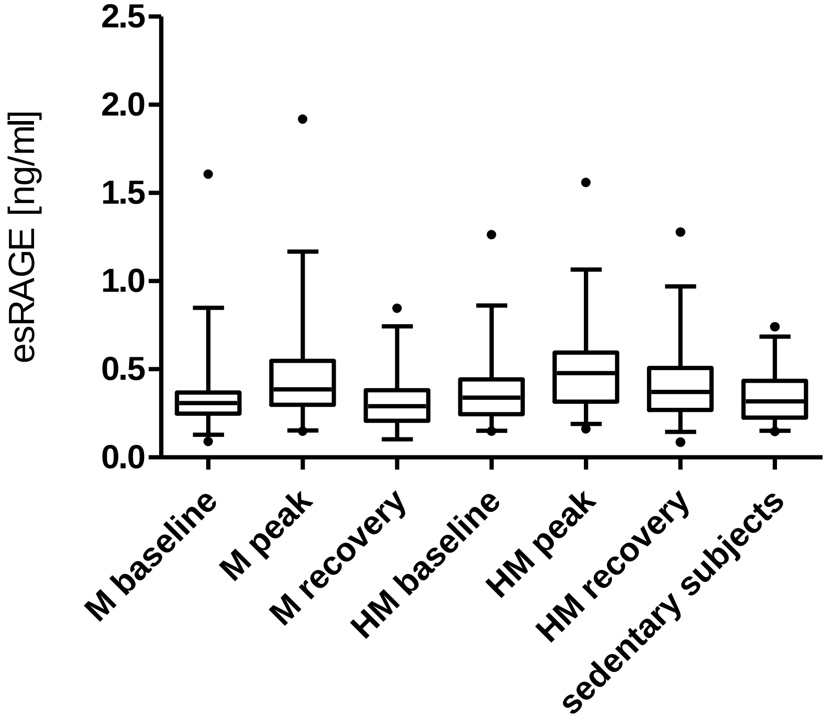

2.5
2.0
1.5
1.0
0.5
0.0
esRAGE [ng/ml]
M baseline
M peak
M recovery
HM baseline
HM peak
HM recovery
sedentary subjects

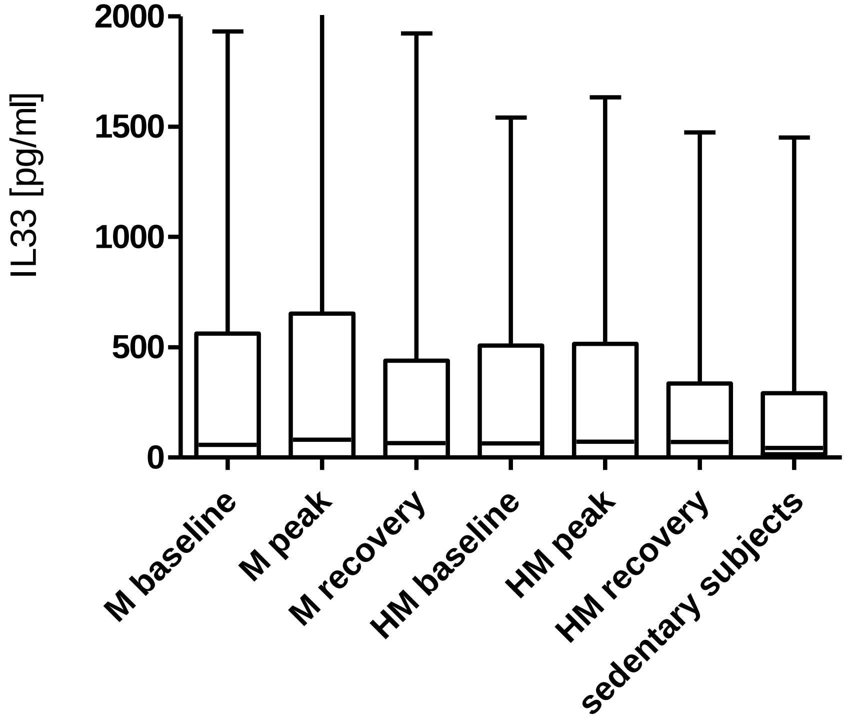

2000
1500
1000
500
0
IL33 [pg/ml]
M baseline
M peak
M recovery
HM baseline
HM peak
HM recovery
sedentary subjects

Fig. Endogenous secretory RAGE in marathoners and half-marathoners at baseline, peak and recovery compared to sedentary controls. Endogenous secretory RAGE remained stable in M and HM runners. M, marathon; HM, half-marathon; baseline, 1-2 days before the run; peak, immediately after the run in the finishing area; recovery, after 2-7 days of recovery.

Fig. Interleukin 33 in marathoners and half-marathoners at baseline, peak and recovery compared to sedentary controls. Interleukin 33 remained stable in M and HM runners. M, marathon; HM, half-marathon; baseline, 1-2 days before the run; peak, immediately after the run in the finishing area; recovery, after 2-7 days of recovery.
